# From blood to bowel: correlating serological markers with mucosal healing and inflammation in patients with inflammatory bowel disease

**DOI:** 10.3389/fimmu.2026.1903539

**Published:** 2026-09-03

**Authors:** Yi Zhang, Zecheng Zhang, Huiling Sun, Bo Diao, Dandan Ma, Dan Wang, Jiankun Jia, Weidong Jin, Haohao Huang

**Affiliations:** 1 Department of General Surgery, General Hospital of Central Theater Command of Chinese People’s Liberation Army (PLA) Graduate Joint Training Base, School of Medicine, University of Science and Technology, Wuhan, Hubei, China; 2 Hubei University of Medicine, Shiyan, Hubei, China; 3 General Hospital of Central Theater Command and Hubei Key Laboratory of Central Nervous System Tumor and Intervention, Wuhan, Hubei, China; 4 Department of Neurosurgery, General Hospital of Central Theater Command of Chinese People’s Liberation Army (PLA) Graduate Joint Training Base, School of Medicine, University of Science and Technology, Wuhan, Hubei, China; 5 Hubei Provincial Clinical Medical Research Center for Minimally Invasive Treatment of Cerebrovascular Diseases, Wuhan, Hubei, China

**Keywords:** Crohn’s disease, inflammation, mucosal healing, serologic indicators, ulcerative colitis

## Abstract

**Background:**

Inflammatory bowel disease (IBD) is a systemic disease characterized by chronic inflammation of the gastrointestinal tract. Serological testing has the advantages of wide sources and easy detection.

**Objective:**

To explore the correlation between the serological markers and endoscopic mucosal healing and histological healing. We also explored the predictive value of individual and combined detection on the disease activity of ulcerative colitis (UC) and Crohn’s disease (CD) and provide reference for clinical non-invasive evaluation.

**Methods:**

We retrospectively analyzed 267 IBD patients treated at our hospital from May 2020 to June 2024. Patients were classified based on endoscopic, pathological, or imaging results into UC (141 patients) and CD (126 patients). UC patients were further divided into mucosal healing (32 cases, IMES ≤ 1), mild (41 cases, 3 ≤ IMES ≤ 5), moderate (49 cases, 6 ≤ IMES ≤ 10), and severe (19 cases, 11 ≤ IMES ≤ 12) phases by the Improved Mayo Endoscopic Score (IMES). CD patients were categorized by the Crohn’s Disease Activity Index (CDAI). According to the Geboes score, patients were divided into a histological healing group (n=63) and a histological activity group (n=204). Pearson analysis examined correlations and ROC analysis assessed their predictive value.

**Results:**

In severe cases, ALB and PA were lower, and CRP, ESR, NLR, and PLR were higher than in moderate cases (P<0.05). The serum ALB and PA levels in the histological activity group were lower, but CRP, ESR, NLR, and PLR were higher than the histological healing group (all P<0.05). Pearson analysis revealed negative correlations between IMES/CDAI scores and ALB/PA levels, and positive correlations with CRP, ESR, NLR, and PLR levels (P<0.05). The combined detection of serological indicators for predicting UC inflammatory activity had an AUC of 0.930, with 88.89% sensitivity and 89.47% specificity, outperforming individual indicators (Z-values all P<0.05). For CD, the combined AUC was 0.906, with 86.75% sensitivity and 85.71% specificity, again surpassing single indicators (Z-values all P<0.05).

**Conclusion:**

Differences in serum ALB, PA, CRP, ESR, NLR, PLR were correlated with mucosal healing and inflammation in IBD patients. Combined detection offers clinical value in predicting disease activity.

## 1 Introduction

Inflammatory bowel disease (IBD) is a chronic inflammation of the gastrointestinal tract that mainly includes two subtypes: ulcerative colitis (UC) and Crohn’s disease (CD). IBD may have a certain risk of deterioration, including narrowing, fistulas, abscesses, etc., affecting the patient’s life and health (1). Due to its complex and unclear etiology, IBD is generally believed to be related to various factors such as genetic susceptibility, environmental factors, immune response, and gut microbiota (2). At present, treatment for IBD mainly focuses on drug therapy, including traditional drugs and biologics. Biological agents such as anti-tumor necrosis factor-α (TNF-α) antibodies have significant effects in controlling inflammation. However, there are still some patients who have primary non-response or secondary loss of response, and further optimization of individualized treatment plans is required. The precise adjustment of the treatment plan depends on an accurate assessment of the degree of inflammation in the disease. Therefore, timely evaluation of the degree of inflammation in IBD is beneficial for timely diagnosis and treatment, thereby improving the prognosis of patients.

Mucosal injury is a common complication of IBD. Study has shown that at the histological level, even when mucosal healing is achieved endoscopically, some patients still have persistent histological inflammation. Histological healing is associated with a lower recurrence rate and hospitalization rate. Therefore, combining endoscopic assessment with histological assessment is helpful for a more comprehensive evaluation of the mucosal condition of patients with IBD (3). However, endoscopy is an effective method for diagnosing and managing IBD, but it is expensive and invasive, which may cause pain and discomfort to patients, and even carry the risk of perforation and bleeding (4). In this regard, serological testing has the advantages of wide sources and easy detection. It can provide non-invasive evaluation of patients based on indicator levels, thereby guiding decision-making at different stages of the disease (5). Among them, C-reactive protein (CRP) and erythrocyte sedimentation rate (ESR) are important traditional inflammatory markers that can reflect the activity level of inflammatory response in the body. Moreover, CRP is widely used as a marker of inflammation due to its rapid rise during the acute-phase response (6). On the other hand, ESR reflects the inflammatory state by measuring the sinking speed of red blood cells in plasma, and its increase also indicates the presence of an inflammatory response in the body (7). In addition, ratio of platelets/lymphocytes (PLR) have recently received widespread attention as an emerging inflammatory indicator. PLR can reflect the immune inflammatory status of the body by calculating the ratio of platelet count to lymphocyte count. Research has shown that PLR exhibits a significant correlation with disease activity in various inflammatory diseases (8). Fecal calprotectin is the most well-documented non-invasive, disease-specific biomarker for IBD. Calprotectin is a calcium- and zinc-binding protein derived from neutrophils, which is released into the intestinal lumen by activated neutrophils during intestinal inflammation and excreted with feces (9). Fecal calprotectin levels are closely associated with the degree of endoscopic mucosal inflammation and histological activity and have been incorporated into China’s guidelines for the diagnosis and treatment of IBD (10). In recent years, the monitoring of serological indicators has attracted widespread attention in the diagnosis of inflammatory diseases, but there is a lack of systematic analysis regarding the specific cutoff values for combined multi-marker testing and their differential predictive performance in UC and CD subtypes (11). In addition, the predictive efficacy comparison of inflammatory ratio indicators such as PA, NLR, and PLR in UC and CD is also insufficient. Based on previous literature, this study systematically compared the expression differences of six serological indicators, ALB, PA, CRP, ESR, NLR, and PLR, in UC and CD subtypes within the same IBD cohort for the first time. The specific cutoff values for each indicator to distinguish between active phase and mucosal healing phase were reported, and the ROC curve was used to quantify the predictive power of the combined detection compared to a single indicator. The aim was to provide a more operational reference for non-invasive evaluation of IBD disease activity in clinical practice.

Based on this, the main objective of this study was: (1) to analyze the expression differences of six serological indicators (ALB, PA, CRP, ESR, NLR, PLR) in patients with different disease activity levels in UC and CD. (2) To explore the correlation between the above indicators and endoscopic mucosal healing and histological healing. (3) To report the specific cutoff values of each indicator, to distinguish between the active period and mucosal healing period, quantify the predictive power of combined detection compared to a single indicator. (4) To provide a more operational reference for non-invasive evaluation of IBD disease activity using serological indicators in clinical practice.

## 2 Materials and methods

### 2.1 Study objects

**Figure 1:**
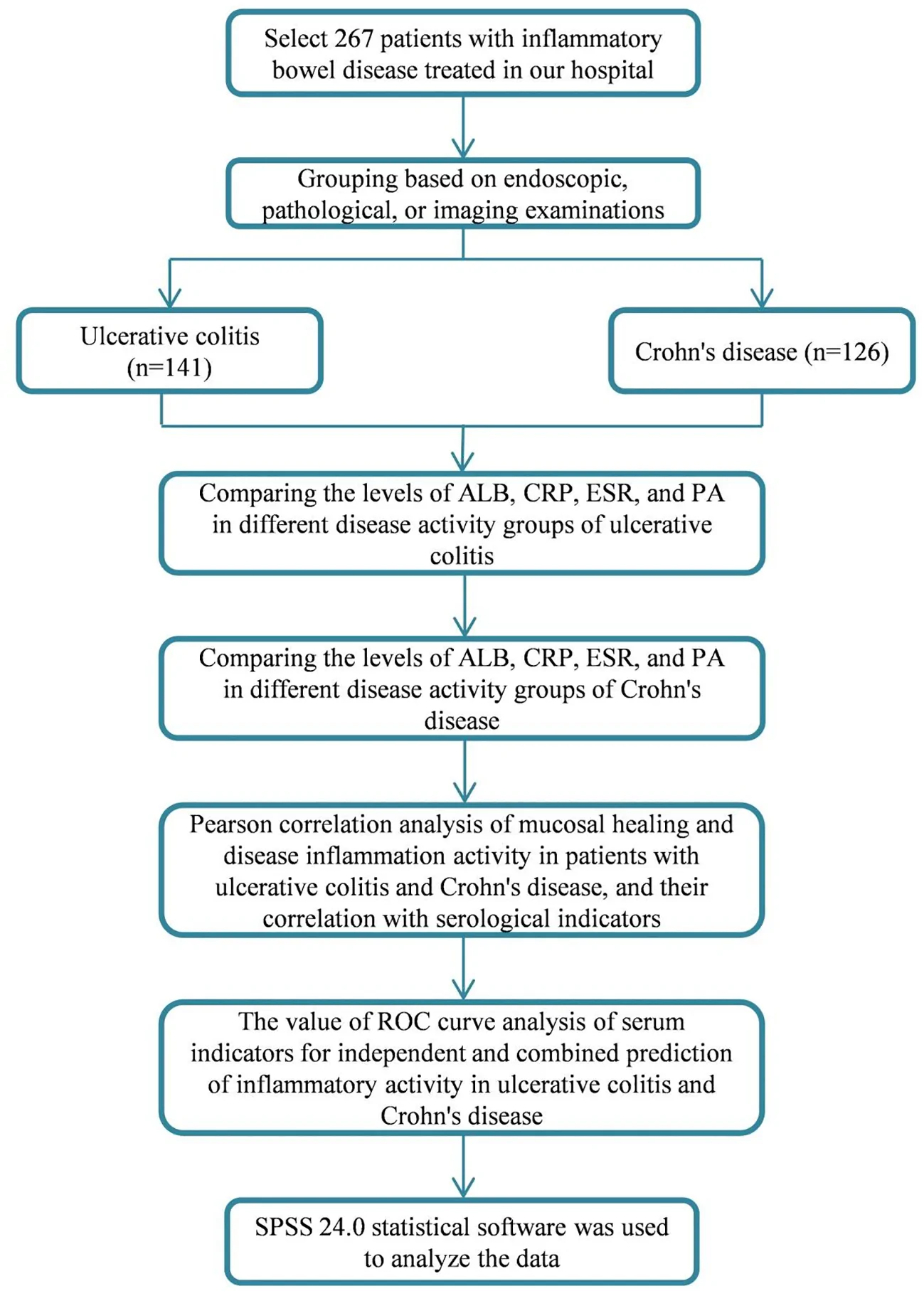
Research flowchart.

A total of 267 IBD patients treated in our hospital during May 2020 to June 2024 were retrospectively selected as the study subjects. Based on the endoscopic, pathological or imaging examinations, these subjects were categorized into UC (141 cases) and CD (126 cases). The average age of patients with UC was 43.49 ± 7.28 years, with 80 males and 61 females. The average body mass index (BMI) was 20.84 ± 2.08 kg/m^2^. The average age of CD patients was 42.52 ± 5.14 years, with 73 males and 53 females, and an average BMI of 19.85 ± 2.26 kg/m^2^. There was no significant statistical difference in age, gender, BMI between patients with UC and CD (*P*>0.05). The Research Flowchart was summarized in **Figure 1**.

The diagnostic criteria for UC (12) were as follows:

1. Typical endoscopic manifestations: The lesion started from the rectum and was diffuse and continuous. The mucosa was extensively affected, with the vascular texture disappearing, the mucosa becoming rough or granular, and multiple ulcers and pseudo-polyps were observed.
2. Typical pathological features: During the active stage, diffuse inflammatory cell infiltration throughout the mucosal layer, crypt abscesses, and a significant reduction in goblet cells could be observed. During the remission period, irregular arrangement (twisting or branching) and atrophy of glands were observed.
3. Typical imaging manifestations: granular changes on the mucosal surface, ulcer formation, pseudo-polyps, and intestinal deformation.

The diagnostic criteria for CD (13) were as follows:

1. Typical endoscopic findings: The lesion presented as segmental and jumping, with scattered patchy ulcers visible in the terminal ileum, and multiple longitudinal ulcers and cobblestone like changes visible in the colon.
2. Typical pathological features: fissure-like ulcers, non-caseous granulomas and transmural inflammation.
3. Typical imaging features: pebble-like changes, fistula formation, abscesses and stenosis.

Inclusion criteria: (1) UC patients met the diagnostic and treatment criteria for UC (12); (2) CD patients met the diagnostic and treatment criteria for CD (13); (3) The patient’s clinical medical records were complete. Exclusion criteria: (1) Patients who had used nonsteroidal anti-inflammatory drugs, immunosuppressants, or other medications within the past 3 months prior to participating in the study; (2) Individuals with combined autoimmune diseases; (3) Patients with combined heart disease or abnormal liver and kidney function; (4) Patients with a history of gastrointestinal or colon resection surgery. This study was a retrospective cross-sectional study, and the data were obtained from the hospital’s electronic medical record system and the laboratory database. This study was ratified by the hospital Ethics Committee.

### 2.2 Grouping criteria

UC: According to the Improved Mayo Endoscopic Score (IMES) (14), patients with UC were divided into three groups based on their disease activity: 32 cases in the mucosal healing phase (IMES ≤ 1), 41 cases in the mild phase (3 ≤ IMES ≤ 5), 49 cases in the moderate phase (6 ≤ IMES ≤ 10), and 19 cases in the severe phase (11 ≤ IMES ≤ 12)]. A score of ≤ 2 was considered the remission period group, and a score of ≥ 3 was considered the active period group.

CD: According to the Crohn’s Disease Activity Index (CDAI) score (15), CD patients were divided into three groups based on their disease activity levels: 31 cases in the mucosal healing stage group (CDAI ≤ 150), 34 cases in the mild stage group (150 ≤ CDAI ≤ 220), 47 cases in the moderate stage group (221 ≤ CDAI ≤ 450), and 14 cases in the severe stage group (CDAI>450)].

### 2.3 Serological factor detection

A total of 4ml of elbow vein blood was collected from each study subjects on an empty stomach in the morning and placed in a vacuum anticoagulant collection tube. The blood sample was centrifuged at 4000 r/min for 15 minutes using a low-temperature high-speed centrifuge (Beijing Pumeijing Co., Ltd., model: SIGMA 1-14K), and the supernatant was collected. All serological indicator tests were completed within 2 hours after specimen collection. Before the tests, the matching quality control products were used for calibration to ensure that the results were within the control range. A blood analyzer (Shanghai Jumu Medical Equipment Co., Ltd., model: MEK-7222K) was used to detect lymphocyte count, platelet count, neutrophil count levels and calculate PLR and NLR levels. A fully automated biochemical analyzer (Shanghai Dibosi Biotechnology Co., Ltd., model: PUZS-300) was adopted to detect the levels of ALB, CRP, ESR, and PA.

### 2.4 Outcome measures

(1) The changes in serum related indicators in patients with UC and CD with different levels of inflammatory activity were detected. (2) Pearson correlation test was used to analyze the correlation between mucosal healing, disease inflammation activity, and serological indicators in patients with UC and CD. (3) Endoscopic mucosal healing assessment: UC was evaluated using the modified Mayo endoscopic score (IMES), while CD was assessed using the CDAI score. All endoscopic examinations were conducted jointly by two experienced digestive endoscopy physicians. (4) Histological assessment: All intestinal mucosal biopsy specimens were fixed with 10% formaldehyde, embedded in paraffin, and stained with HE. Two pathologists independently evaluated them using the Geboes scoring system. The Geboes scoring system (16) assessed six histological features: structural changes (grade 0), chronic inflammatory cell infiltration (grade 1), eosinophils in the lamina propria (grade 2A), neutrophils (grade 2B), epithelial neutrophils (grade 3), crypt destruction (grade 4), erosion or ulceration (grade 5). Each grade was divided into 0 to 3 subgrades, with a total score ranging from 0 to 5.4 points. The higher the score, the more severe the histological inflammation. Histological healing was defined as a Geboes score of less than 2.0. When the opinions of two pathologists were inconsistent, consensus was reached through consultation. The changes in serological related indicators of patients with UC and CD with different degrees of inflammatory activity were detected.

### 2.5 Statistical analysis

SPSS 24.0 statistical software was used to analyze the data. In this study, the measurement data (NLR, PLR, ALB, CRP, ESR, etc.) were found to follow a normal distribution. Independent sample t-test was used for comparison between two groups, one-way analysis of variance was used for multiple groups, and the least significant difference (LSD) test was used for pairwise comparison, all expressed as ( $\bar{x}$ ± *s*). The comparison of enumeration data between groups in the study was conducted using a *χ^2^* test, expressed as [cases (%)]. Correlation tests were conducted using Pearson correlation analysis. ROC curve analysis was used to evaluate the independent and combined predictive value of serologic indices for inflammation activity in UC and CD. The statistical results indicated that a difference of P<0.05 was statistically significant.

## 3 Results

### 3.1 Clinical characteristics of 267 patients with inflammatory bowel disease

There was no significant difference in age, gender, and BMI between the two groups of patients (*P > 0.05*). The onset time of the disease in the UC group was longer than that in the CD group (*P < 0.05*). In terms of treatment, the proportion of using 5-aminosalicylic acid preparations in the UC group was higher than that in the CD group (*P < 0.05*). The proportion of using immunosuppressants, biologics and glucocorticoids in the CD group was higher than that in the UC group (*P < 0.05*, **Table 1**).

**Table 1:** Clinical characteristics of 267 patients with inflammatory bowel disease [( $\bar{x}$ ± *s*), cases (%)].

Clinical features	Ulcerative colitis group (UC, n = 141)	Crohn’s disease group (CD, n = 126)	t/χ²/Z	P value
Age (years)	43.49 ± 7.28	42.52 ± 5.14	1.240	0.216
Gender			0.040	0.842
Male	80 (56.74)	73 (57.94)		
Female	61 (43.26)	53 (42.06)		
BMI (kg/m²)	20.84 ± 2.08	19.85 ± 2.26	3.719	<0.001
Disease onset time [months]	24.0 (12.0, 48.0)	18.0 (9.0, 36.0)	-2.214	0.027
Previous treatment history				
5-aminosalicylic acid preparation	98 (69.50)	32 (25.40)	54.372	<0.001
Immunosuppressant	31 (21.99)	45 (35.71)	6.498	0.011
Biological agents	12 (8.51)	49 (38.89)	34.730	<0.001
Glucocorticoid	26 (18.44)	38 (30.16)	5.077	0.024
Lesion area (UC)				
Rectum/Sigmoid colon	52 (36.88)	—		
Left half of colon	41 (29.08)	—		
Extensive colon	48 (34.04)	—		
Lesion site (CD)				
Terminal ileum	—	38 (30.16)		
Colon	—	42 (33.33)		
Ileocolon	—	46 (36.51)		

### 3.2 Comparison of serological indicators of different activity levels in UC

There had significant differences in various indicators among the mucosal healing period group, mild period group, moderate period group, and severe period group (*P* < 0.05). The serum levels of ALB and PA in the mild, moderate, and severe stages were lower than those in the mucosal healing stage group, while the levels of CRP, ESR, PLR, and NLR were higher than those in the mucosal healing stage group (*P* < 0.05). Compared with the mild stage group, serum ALB and PA levels were lower and CRP, ESR, NLR, and PLR levels were higher in the moderate and severe stage groups (*P* < 0.05). Compared with the moderate stage group, serum ALB and PA levels were lower and CRP, ESR, NLR, and PLR levels were higher in the severe stage group (*P* < 0.05, **Table 2**). As the disease activity of UC progresses from the mucosal healing stage to the severe stage, the levels of ALB and PA showed a gradually decreasing trend, while the levels of CRP, ESR, NLR and PLR showed a gradually increasing trend (**Table 2**).

**Table 2:** Comparison of serological indicators of different activity levels in UC ( $\bar{x}$ ± *s*).

Groups	Mucosal healing period group (n=32)	Mild period group (n=41)	Moderate period group (n=49)	Severe period group (n=19)	*F*	*P*
ALB (g/L)	46.45 ± 1.25	44.16 ± 2.52^*^	40.74 ± 3.88^*#^	34.49 ± 4.30^*#△^	66.23	<0.001
CRP (mg/L)	0.05 ± 0.01	0.79 ± 0.23^*^	3.02 ± 1.02^*#^	4.23 ± 1.75^*#△^	138.23	<0.001
ESR (mm/h)	6.65 ± 1.20	13.49 ± 3.25^*^	34.89 ± 12.66^*#^	67.58 ± 23.17^*#△^	141.07	<0.001
NLR	1.05 ± 0.20	1.68 ± 0.52^*^	3.57 ± 1.02^*#^	6.23 ± 1.30^*#△^	198.65	<0.001
PA (mg/L)	312.38 ± 35.85	284.46 ± 36.50^*^	202.49 ± 56.25^*#^	82.58 ± 25.12^*#△^	139.85	<0.001
PLR	94.38 ± 21.27	136.29 ± 27.24^*^	241.73 ± 26.71^*#^	423.24 ± 51.32^*#△^	566.47	<0.001

^*^P < 0.05 compared with mucosal healing period; ^#^*P* < 0.05 compared with the mild period; ^△^*P* < 0.05 compared with the moderate period.

### 3.3 Comparison of serological indicators in different histological activity groups

All patients were divided into the histological healing group (Geboes score < 2.0, n = 63) and the histological activity group (Geboes score ≥ 2.0, n = 204) based on the Geboes score. The serum levels of ALB and PA in the histological activity group were lower than those in the histological healing group, while the levels of CRP, ESR, NLR, and PLR were higher than those in the histological healing group. The differences between the groups were statistically significant (all *P < 0.05*). The comparison of serological indicators between the two groups was shown in **Table 3**.

**Table 3:** Comparison of serological indicators for different histological activity groups.

Indicators	Histological healing group (n=63)	Histological activity group (n = 204)	t	P
ALB (g/L)	45.38 ± 3.67	38.52 ± 5.21	9.863	<0.001
PA (mg/L)	308.52 ± 41.27	178.45 ± 62.38	15.824	<0.001
CRP (mg/L)	0.08 ± 0.03	2.85 ± 1.52	15.014	<0.001
ESR (mm/h)	7.12 ± 2.05	38.52 ± 18.34	13.672	<0.001
NLR	1.28 ± 0.35	4.18 ± 1.82	12.938	<0.001
PLR	112.38 ± 28.64	295.46 ± 118.52	12.406	<0.001

### 3.4 The correlation between mucosal healing and disease inflammation activity of UC and serological indicators

Pearson correlation analysis confirmed that the IMES score and CDAI score were negatively correlated with serum ALB and PA levels, and positively correlated with serum CRP, ESR, NLR and PLR levels (*P* < 0.05, **Table 4**).

**Table 4:** The correlation between mucosal healing and disease inflammation activity of UC and serological indicators.

Indicators	IMES score	
	*R*	*P*
ALB	-0.699	<0.05
CRP	0.632	<0.05
ESR	0.791	<0.05
NLR	0.639	<0.05
PA	-0.730	<0.05
PLR	0.696	<0.05

### 3.5 Comparison of serological indicators of different activity levels in CD

As the disease activity of CD progresses from the mucosal healing stage to the severe stage, the levels of ALB and PA were gradually decreased, but the levels of CRP, ESR, NLR, and PLR were gradually increased. There had significant differences in various indicators among the mucosal healing period group, mild period group, moderate period group, and severe period group (*P* < 0.05). The serum levels of ALB and PA in the mild, moderate, and severe stages were lower than those in the mucosal healing stage group, while the levels of CRP, ESR, PLR, and NLR were higher than those in the mucosal healing stage group (*P* < 0.05). Compared with the mild stage group, serum ALB and PA levels were lower and CRP, ESR, NLR, and PLR levels were higher in the moderate and severe stage groups (*P* < 0.05). Compared with the moderate stage group, serum ALB and PA levels were lower and CRP, ESR, NLR, and PLR levels were higher in the severe stage group (*P* < 0.05, **Table 5**).

**Table 5:** Comparison of serological indicators of different activity levels in CD ( $\bar{x}$ ± *s*).

Groups	Mucosal healing period group (n=31)	Mild period group (n=34)	Moderate period group (n=47)	Severe period group (n=14)	*F*	*P*
ALB(g/L)	45.69 ± 2.40	42.16 ± 3.63^*^	39.28 ± 2.44^*#^	34.57 ± 4.23^*#△^	49.09	<0.001
CRP(mg/L)	0.10 ± 0.03	1.37 ± 0.41^*^	2.10 ± 0.68^*#^	5.19 ± 1.38^*#△^	194.02	<0.001
ESR(mm/h)	7.25 ± 2.13	13.53 ± 3.16^*^	30.18 ± 8.35^*#^	64.36 ± 22.85^*#△^	142.61	<0.001
NLR	1.52 ± 0.46	2.70 ± 0.78^*^	5.24 ± 1.40^*#^	6.27 ± 1.96^*#△^	93.99	<0.001
PA(mg/L)	329.68 ± 40.29	264.16 ± 51.26^*^	174.46 ± 40.49^*#^	79.49 ± 3.25^*#△^	155.78	<0.001
PLR	128.17 ± 30.63	201.24 ± 24.62^*^	294.84 ± 50.61^*#^	513.15 ± 118.26^*#△^	187.55	<0.001

^*^P < 0.05 compared with mucosal healing period; ^#^*P* < 0.05 compared with the mild period; ^△^*P* < 0.05 compared with the moderate period.

### 3.6 The correlation between mucosal healing and disease inflammation activity of CD with serological indicators

Pearson correlation analysis confirmed that the CDAI score was negatively correlated with serum ALB and PA levels, and positively correlated with serum CRP, ESR, NLR and PLR levels (*P* < 0.05, **Table 6**).

**Table 6:** The correlation between mucosal healing and disease inflammation activity of CD and serological indicators.

Indicators	CDAI score	
	*r*	*P*
ALB	-0.553	<0.05
CRP	0.585	<0.05
ESR	0.829	<0.05
NLR	0.678	<0.05
PA	-0.762	<0.05
PLR	0.806	<0.05

### 3.7 The value of independent and combined serological indicators in predicting the inflammatory activity of UC

**Figure 2:**
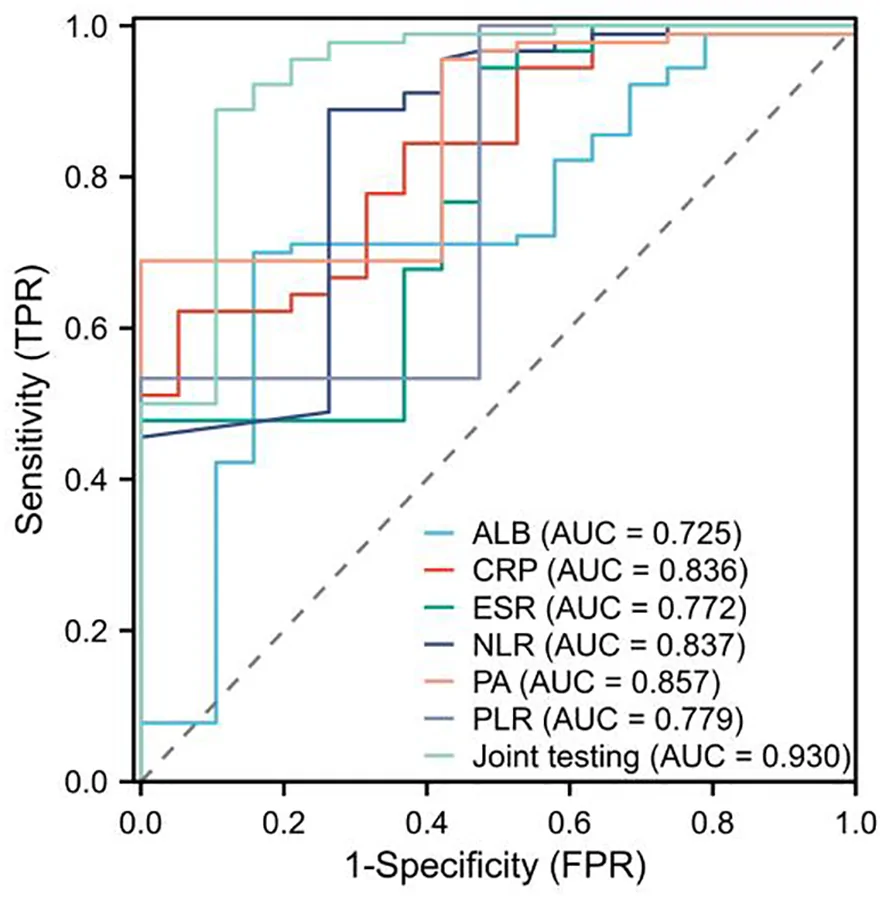
ROC curve of independent and combined prediction of inflammatory activity in UC by serological indicators.

The ROC curve analysis showed that the AUC for predicting the inflammatory activity of UC by combined detection of serological indicators was 0.930, with a sensitivity of 88.89% and a specificity of 89.47%. The AUC of combined detection was much higher than that of single detection of each indicator and had certain clinical application value (Z = 3.326, 2.441, 3.685, 2.440, 2.269, 3.690, *P* < 0.05, **Table 7**, **Figure 2**).

**Table 7:** ROC curve of independent and combined prediction of inflammatory activity in UC by serological indicators.

Indicator	AUC	95%CI	Sensitivity	Specificity	Cut-off value	Youden’s index
ALB	0.725	0.591-0.858	70.00	84.21	40.94 g/L	0.542
CRP	0.836	0.748-0.925	62.22	94.74	2.36 mg/L	0.570
ESR	0.772	0.652-0.892	47.78	92.31	48.75 mm/h	0.401
NLR	0.837	0.729-0.945	88.89	73.68	4.41	0.626
PA	0.857	0.776-0.938	70.89	93.01	102.13 mg/L	0.639
PLR	0.779	0.661-0.897	53.33	90.47	249.7	0.438
Combined detection	0.930	0.858-0.989	88.89	89.47	/	0.784

### 3.8 The value of independent and combined serological indicators in predicting the inflammatory activity of CD

**Figure 3:**
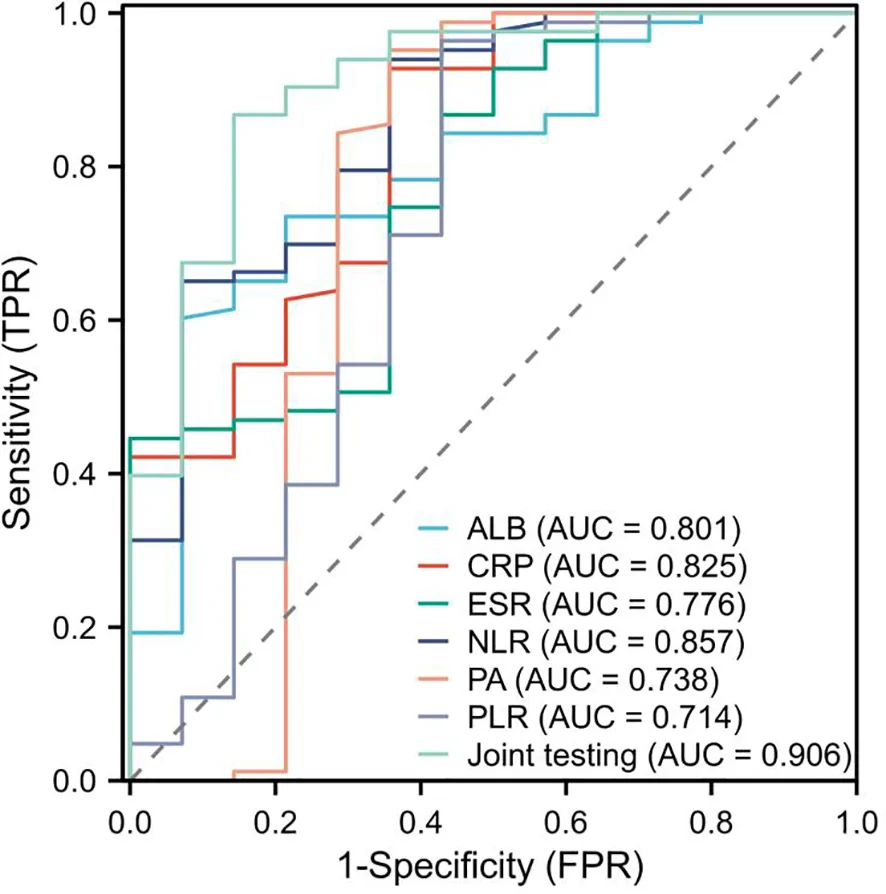
ROC curve of independent and combined prediction of inflammatory activity in CD by serological indicators.

The AUC for predicting the inflammatory activity of CD using combined serologic indices was 0.906, with a sensitivity of 86.75% and a specificity of 85.71%. The AUC of the combined detection was much higher than that of each individual indicator, indicating certain clinical application value (Z = 2.873, 2.659, 3.687, 3.115, 4.021, all *P* < 0.05, **Table 8**, **Figure 3**).

**Table 8:** The value of independent and combined serological indicators in predicting the inflammatory activity of CD analyzed by ROC curve.

Indicators	AUC	95% CI	Sensitivity	Specificity	Cut-off value	Youden’s index
ALB	0.801	0.676-0.897	60.24	92.86	37.65g/L	0.531
CRP	0.825	0.698-0.902	92.77	64.29	3.98mg/L	0.571
ESR	0.776	0.639-0.818	58.58	90.20	50.34mm/L	0.488
NLR	0.857	0.743-0.902	93.98	64.29	6.18	0.583
PA	0.738	0.519-0.821	95.18	64.29	83.65mg/L	0.595
PLR	0.714	0.520-0.908	96.39	57.14	487.19	0.535
Combined detection	0.906	0.813-0.957	86.75	85.71	/	0.725

## 4 Discussion

Inflammatory bowel disease (IBD) is a chronic recurrent gastrointestinal inflammatory disease, and endoscopic examination is the gold standard for evaluating its mucosal healing status, but the application is limited in long-term follow-up due to its invasiveness and high cost. IBD is a chronic recurrent inflammatory disease of the gastrointestinal tract. Many factors, including immune abnormalities, genetic factors, dietary changes, host microbial homeostasis, may be related to the increase in the incidence rate of IBD (17). Mucosal healing is a wound repair process that can restore the integrity and balance function of the damaged epithelial barrier after its integrity is disrupted (18). Previous studies have confirmed that mucosal healing can reduce the recurrence rate, hospitalization rate, and surgical resection rate (19). In recent years, mucosal healing has been recognized as one of the core goals in the treatment of CD, and its significance has surpassed that of mere clinical remission (20).

Serological indicators ALB, PA, CRP, ESR, NLR, and PLR are important indicators for evaluating the nutritional status and disease severity of patients with IBD. Previous studies have found that about 20% to 85% of patients with inflammatory diseases may be accompanied by malnutrition, which may be related to further exacerbation of inflammatory reactions caused by gastrointestinal dysfunction, impaired nutrient absorption, and immune function (21). ALB, PA, and other indicators can be used to reflect the nutritional status of the body. ALB is a non-specific transporter protein that is a factor in regulating the volume of intravascular space. One of the main reasons for the decrease in ALB is that intestinal inflammation causes plasma proteins to leak out from the damaged intestinal mucosa, thereby leading to hypoproteinemia (22). In addition, ALB can also serve as a negative acute phase protein, and its level is directly affected by the severity of acute infection. According to reports, ALB levels have high sensitivity and specificity for CD (23). PA, also known as thyroid hormone transporter protein, has a shorter half-life than ALB, allowing PA to more sensitively reflect changes in liver synthesis function (24). In a study of UC, it was found that PA levels gradually decreased as the condition worsened. The level of PA is negatively correlated with the severity of the disease, and it has certain value in predicting the activity of UC (25). In our study, the Geboes score was used to assess the degree of histological activity. The results showed that the levels of CRP, ESR, NLR, and PLR in the histological activity group (Geboes score ≥ 2.0) were higher than those in the histological healing group (Geboes score < 2.0), while the levels of ALB and PA were lower in the histological activity group. These results suggested that serological indicators were not only related to endoscopic mucosal healing but also had an association with the degree of histological activity under the microscope. Moreover, the results of this study found that the levels of serum ALB and PA in patients with mucosal healing were significantly lower than those in patients with active stage, and the levels of serum ALB and PA were negatively correlated with IMES score and CDAI score. The above results indicated that serum ALB and PA levels could serve as indicators reflecting the severity of inflammatory bowel disease. The possible reason for this might be that in inflammatory bowel disease, inflammation damage to the intestinal mucosa and disruption of intestinal barrier function lead to increased intestinal permeability, causing serum ALB to leak from blood vessels into tissue gaps, resulting in a decrease in serum ALB levels. Inflammatory factors such as IL-6 can also promote the production of acute phase response proteins in the liver, and the synthesis of these proteins consumes precursor substances of serum ALB, further leading to a decrease in ALB levels (26). PA is synthesized by the liver and belong to negative acute-phase protein. Its synthesis is inhibited under inflammatory conditions. At the same time, intestinal inflammation and nutrient absorption disorders lead to insufficient protein intake, further reducing the level of PA (27). The half-life of PA is approximately 2 days, which is much shorter than that of ALB (about 20 days). Therefore, PA can reflect changes in the body’s nutritional status and inflammatory stress more promptly and sensitively (27).

CRP, ESR, NLR, and PLR are important serological indicators reflecting systemic inflammatory response. The changes of their levels are closely related to the disease activity of IBD (28–31). CRP is a positive acute phase reactant commonly used to evaluate inflammatory processes. Previous studies have found that inflammatory reactions may affect the synthesis of ALB. The excessive release of inflammatory mediators can cause a large deposition of extracellular matrix in the intestine, which leads to fibrous narrowing of the intestine, affecting the absorption of nutrients and further exacerbating disease development (28). According to reports, CRP/ALB has certain predictive value for the severity of UC under endoscopy and can be used as an important tool for evaluating the patient’s condition (29). ESR is one of the most classic inflammatory markers. Research has found that ESR can help evaluate chronic and subacute inflammation. During the recovery period, the rate of ESR downregulation is slower than that of CRP, and combined detection of ESR and CRP may be more helpful in tracking the inflammatory process (30). NLR and PLR are indicators closely related to systemic inflammatory response. Previous studies have found that decreased lymphocytes and increased neutrophils are part of the immune system’s physiological response to systemic inflammation and stress, which can be closely related to the cascade of cytokines secreted and destructive tissues (31). Previous studies have found that lymph function in IBD patients is abnormal at the circulatory and mucosal levels. Additionally, NLR levels may be correlated with total protein (TP) and ALB levels to some extent (32). The results of our study showed that the levels of CRP, ESR, NLR, and PLR were gradually increased as the disease activity worsens. Their levels were positively correlated with the IMES score and the CDAI score (all *P < 0.05*). Therefore, detecting various serological indicators is beneficial for early assessment of patients’ disease inflammation activity, thereby screening suspected patients for further diagnosis and treatment. The main contributions of this study compared to previous literature are as follows. Firstly, it provides specific cutoff values for six serological indicators to distinguish the active and mucosal healing phases in the two subtypes of UC and CD. Although previous studies have confirmed that indicators such as NLR and PLR are related to the disease activity of IBD, they mostly did not provide quantitative thresholds that can directly guide clinical decisions (8, 33). This study separately reported the specific cutoff values for indicators such as ALB (UC: 40.94 g/L; CD: 37.65 g/L), PA (UC: 102.13 mg/L; CD: 83.65 mg/L), and NLR (UC: 4.41; CD: 6.18). Secondly, the differences in predictive efficacy of the same indicators in UC and CD were systematically compared. In this study, the AUC of the combined detection in UC was 0.930, and in CD it was 0.906. This suggested that the serological indicators have a slightly better ability to distinguish the activity of UC compared to CD. This is consistent with the trend reported in the literature that fecal calprotectin has a better diagnostic efficacy in UC than in CD (34). Thirdly, the added value of combined detection compared to a single indicator was quantified. In this study, the AUC of the combined detection was 0.073 - 0.205 (UC) and 0.049 - 0.192 (CD) higher than that of a single indicator, suggesting that the combination of multiple indicators can enhance the ability to distinguish disease activity. These findings have expanded the conclusions of previous single-indicator correlation studies, providing a more practical reference basis for the clinical application of serological indicators in the assessment of IBD conditions.

ROC curve analysis can quantitatively assess the predictive value of serological indicators for the disease activity of IBD. The ROC curve analysis results of our study showed that the AUC for predicting the inflammatory activity of UC by combining serological indicators was 0.930, with a sensitivity of 88.89% and a specificity of 89.47%. The AUC for predicting the inflammatory activity of CD using a combination of serological indicators was 0.906, with a sensitivity of 86.75% and a specificity of 85.71%. The above results indicated that there was a correlation between serological indicators and IBD disease activity, which can be used as a reference indicator for clinical auxiliary assessment of the disease, but it cannot replace endoscopic examination. Muhanmet et al. (33) found that the average NLR and PLR values of the endoscopic active disease group were higher than those of other groups, and the endoscopic remission group was higher than the control group. They also found that high ESR was an independent predictor of endoscopic active disease. Combining NLR and PLR with ESR and CRP is more accurate in evaluating mucosal disease and inflammation in UC. Fecal calprotectin is currently recognized as a non-invasive biomarker for IBD intestinal inflammation, with different clinical positioning compared to serological indicators. A meta-analysis of 24 prospective studies showed that fecal calprotectin had a combined sensitivity of 85% for assessing endoscopic activity in IBD, and its diagnostic efficacy in UC was superior to that in CD (34). Fecal calprotectin is significantly positively correlated with endoscopic scoring and is an important tool for evaluating mucosal healing. Kawashima et al. found (35) that fecal calprotectin<82 µg/g can predict histological healing in UC patients undergoing endoscopic remission. Compared with systemic inflammatory indicators such as serum CRP and ESR, fecal calprotectin directly originates from the inflammatory sites of the intestinal mucosa and is less affected by extraintestinal factors. Therefore, it has higher specificity in reflecting local intestinal inflammation. Future research can compare or combine fecal calprotectin with serological indicators to further improve the accuracy of IBD disease activity assessment.

Based on the results of this study, the following practical clinical recommendations are proposed: (1) for UC patients with serum ALB<40.94 g/L, PA<102.13 mg/L, NLR>4.41, or CRP>2.36 mg/L, it suggests the presence of active lesions. It is recommended to undergo further endoscopic examination to clarify the condition. (2) For CD patients, when ALB<37.65 g/L, PA<83.65 mg/L, NLR>6.18, or CRP>3.98 mg/L, it also indicates possible disease activity. Such cases should be evaluated comprehensively in conjunction with clinical manifestations. (3) The combined detection of six indicators including ALB, PA, CRP, ESR, NLR, and PLR can significantly enhance the predictive efficacy for disease activity (UC: AUC = 0.930; CD: AUC = 0.906). During the follow-up period when endoscopic examinations cannot be conducted frequently, the serological indicators combined detection can be used as an auxiliary means for disease monitoring. (4) For patients with sustained reductions in ALB and PA, nutritional support therapy should be considered, to improve their nutritional status and immune function. The above suggestions are intended to provide guidance for clinicians when choosing non-invasive monitoring strategies in the long-term management of IBD patients. However, serological indicators cannot replace the gold standard status of endoscopic examination.

In summary, the main objective of this study is to evaluate the correlation between six serological indicators (ALB, PA, CRP, ESR, NLR, and PLR), and endoscopic mucosal healing and disease activity in patients with inflammatory bowel disease, and to quantify the predictive power of combined detection compared to a single indicator. The research has confirmed that these indicators were significantly correlated with mucosal healing and disease inflammatory activity in both UC and CD subtypes. Combined detection had a high predictive value for assessing disease activity. However, serological indicators are greatly influenced by external factors, and their results may have certain biases. Moreover, they still cannot replace the gold standard status of endoscopic examination.

The subsequent research plans are as follows: (1) Include intestinal-specific markers such as fecal calprotectin and conduct comparisons or joint analyses with serological indicators to further improve the accuracy of non-invasive assessment of IBD disease activity. (2) Conduct a prospective, multi-center cohort study to verify the clinical applicability of the cutoff values of each indicator proposed in this study in different populations. (3) Explore the feasibility of incorporating the above serological indicators into the comprehensive scoring system for the activity of IBD, in order to optimize the formulation of individualized treatment plans.

## Data Availability

The original contributions presented in the study are included in the article/supplementary material. Further inquiries can be directed to the corresponding authors.

## References

[1] Bromke MA Neubauer K Kempiński R Krzystek-Korpacka M . Faecal calprotectin in assessment of mucosal healing in adults with inflammatory bowel disease: a meta-analysis. J Clin Med. (2021) 10:2203. doi: 10.3390/jcm10102203 34069684 PMC8161009

[2] Al-Rshaidat MMD Al-Sharif S Refaei AA Shewaikani N Alsayed AR Rayyan YM . Evaluating the clinical application of the immune cells' ratios and inflammatory markers in the diagnosis of inflammatory bowel disease. Pharm Pract (Granada). (2023) 21:2755. doi: 10.18549/pharmpract.2023.1.2755 37090461 PMC10117338

[3] Krzystek-Korpacka M Kempiński R Bromke M Neubauer K . Biochemical biomarkers of mucosal healing for inflammatory bowel disease in adults. Diagnostics (Basel). (2020) 10:367. doi: 10.3390/diagnostics10060367 32498475 PMC7344443

[4] Costa MHM Sassaki LY Chebli JMF . Fecal calprotectin and endoscopic scores: the cornerstones in clinical practice for evaluating mucosal healing in inflammatory bowel disease. World J Gastroenterol. (2024) 30:3022–35. doi: 10.3748/wjg.v30.i24.3022 38983953 PMC11230062

[5] López-Ureña NM Calero-Bernal R González-Fernández N Blaga R Koudela B Ortega-Mora LM . Optimization of the most widely used serological tests for a harmonized diagnosis of Toxoplasma gondii infection in domestic pigs. Vet Parasitol. (2023) 322:110024. doi: 10.1016/j.vetpar.2023.110024 37729831

[6] Kang HW . A novel serum biomarker of endoscopic mucosal healing in inflammatory bowel disease. Intest Res. (2024) 22:3–4. doi: 10.5217/ir.2023.00198 38327002 PMC10850698

[7] Daniluk U Daniluk J Krasnodebska M Lotowska JM Sobaniec-Lotowska ME Lebensztejn DM . The combination of fecal calprotectin with ESR, CRP and albumin discriminates more accurately children with Crohn's disease. Adv Med Sci. (2019) 64:9–14. doi: 10.1016/j.advms.2018.08.001 30237086

[8] Zahmatkesh A Sohouli MH Hosseini SME Rohani P . The role of platelet-to-lymphocyte ratio and neutrophil-to-lymphocyte ratio in the diagnosis and severity of inflammatory bowel disease in children. Eur J Pediatr. (2023) 182:4263–70. doi: 10.1007/s00431-023-05110-0 37458815

[9] Bertani L Mumolo MG Tapete G Albano E Baiano Svizzero G Zanzi F . Fecal calprotectin: current and future perspectives for inflammatory bowel disease treatment. Eur J Gastroenterol Hepatol. (2020) 32:1091–8. doi: 10.1097/meg.0000000000001731 32282400

[10] State M Negreanu L Voiosu T Voiosu A Balanescu P Mateescu RB . Surrogate markers of mucosal healing in inflammatory bowel disease: a systematic review. World J Gastroenterol. (2021) 27:1828–40. doi: 10.3748/wjg.v27.i16.1828 33967560 PMC8072191

[11] Reghefaoui M Peresuodei TS Saavedra Palacios MS Gill A Orji C Reghefaoui T . The role of serological markers in the prediction of disease course and response to therapy in inflammatory bowel disease. Cureus. (2023) 15:e48442. doi: 10.7759/cureus.48442 38073917 PMC10702424

[12] Murphy ME Bhattacharya S Axelrad JE . Diagnosis and monitoring of ulcerative colitis. Clin Colon Rectal Surg. (2022) 35:421–7. doi: 10.1055/s-0042-1758047 36591402 PMC9797286

[13] Evaristo G Szczepanski J Farag MS Rubin DT Campbell LK Marcus VA . Crohn's disease features in anastomotic biopsies from patients with and without Crohn's disease: diagnostic and prognostic value. Mod Pathol. (2023) 36:100325. doi: 10.1016/j.modpat.2023.100325 37660927

[14] Shimodaira Y Watanabe K Fukuda S Watanabe N Koizumi S Matsuhashi T . Limited endoscopic mucosal inflammation on equivalent to Mayo endoscopic subscore of 0 unaffect clinical relapse of ulcerative colitis. Scand J Gastroenterol. (2022) 57:165–8. doi: 10.1080/00365521.2021.1991467 34663142

[15] Ishida N Miyazu T Suzuki T Tamura S Tani S Yamade M . Evaluation of the modified Crohn's disease activity index in patients with Crohn disease with enterostomy: a single-center observational study. Med (Baltimore). (2021) 100:e24717. doi: 10.1097/md.0000000000024717 33578614 PMC10545123

[16] Fabian O Bajer L . Histopathological assessment of the microscopic activity in inflammatory bowel diseases: what are we looking for? World J Gastroenterol. (2022) 28:5300–12. doi: 10.3748/wjg.v28.i36.5300 36185628 PMC9521520

[17] Liu D Saikam V Skrada KA Merlin D Iyer SS . Inflammatory bowel disease biomarkers. Med Res Rev. (2022) 42:1856–87. doi: 10.1002/med.21893 35603998 PMC10321231

[18] Otte ML Lama Tamang R Papapanagiotou J Ahmad R Dhawan P Singh AB . Mucosal healing and inflammatory bowel disease: therapeutic implications and new targets. World J Gastroenterol. (2023) 29:1157–72. doi: 10.3748/wjg.v29.i7.1157 36926666 PMC10011951

[19] Dal Buono A Roda G Argollo M Zacharopoulou E Peyrin-Biroulet L Danese S . Treat to target or 'treat to clear' in inflammatory bowel diseases: one step further? Expert Rev Gastroenterol Hepatol. (2020) 14:807–17. doi: 10.1080/17474124.2020.1804361 32762582

[20] Cucchiara S D'Arcangelo G Isoldi S Aloi M Stronati L . Mucosal healing in Crohn's disease: new insights. Expert Rev Gastroenterol Hepatol. (2020) 14:335–45. doi: 10.1080/17474124.2020.1759416 32315209

[21] Godala M Gaszyńska E Walczak K Małecka-Wojciesko E . Evaluation of albumin, transferrin and transthyretin in inflammatory bowel disease patients as disease activity and nutritional status biomarkers. Nutrients. (2023) 15:3479. doi: 10.3390/nu15153479 37571416 PMC10421392

[22] Schoenefuss F Hoffmann P . Serum γ-globulin and albumin concentrations predict secondary loss of response to anti-TNFα in inflammatory bowel disease patients. Eur J Gastroenterol Hepatol. (2019) 31:1563–8. doi: 10.1097/meg.0000000000001493 31567711

[23] Huang J Lu J Jiang FY Song TJ . Platelet/albumin ratio and plateletcrit levels are potential new biomarkers for assessing endoscopic inflammatory bowel disease severity. BMC Gastroenterol. (2023) 23:393. doi: 10.21203/rs.3.rs-2950900/v1 37964205 PMC10644627

[24] Wang Y Li C Wang W Wang J Li J Qian S . Serum albumin to globulin ratio is associated with the presence and severity of inflammatory bowel disease. J Inflammation Res. (2022) 15:1907–20. doi: 10.2147/jir.s347161 35313674 PMC8933625

[25] Cagnasso F Borrelli A Bottero E Benvenuti E Ferriani R Marchetti V . Comparative evaluation of peripheral blood neutrophil to lymphocyte ratio, serum albumin to globulin ratio and serum C-reactive protein to albumin ratio in dogs with inflammatory protein-losing enteropathy and healthy dogs. Anim (Basel). (2023) 13:484. doi: 10.3390/ani13030484 36766371 PMC9913579

[26] Yagi S Furukawa S Shiraishi K Hashimoto Y Tange K Mori K . Effect of disease duration on the association between serum albumin and mucosal healing in patients with ulcerative colitis. BMJ Open Gastroenterol. (2021) 8:e000662. doi: 10.1136/bmjgast-2021-000662 34099464 PMC8186756

[27] Wu Z Zeng H Zhang L Pu Y Li S Yuan Y . Patchouli alcohol: a natural sesquiterpene against both inflammation and intestinal barrier damage of ulcerative colitis. Inflammation. (2020) 43:1423–35. doi: 10.1007/s10753-020-01219-8 32388657

[28] Chen YH Wang L Feng SY Cai WM Chen XF Huang ZM . The relationship between C-reactive protein/albumin ratio and disease activity in patients with inflammatory bowel disease. Gastroenterol Res Pract. (2020) 2020:3467419. doi: 10.1155/2020/3467419 32655630 PMC7327549

[29] Feng W Zhu L Liu Y Xu L Shen H . C-reactive protein/albumin ratio and IL-6 are associated with disease activity in patients with ulcerative colitis. J Clin Lab Anal. (2023) 37:e24843. doi: 10.1002/jcla.24843 36725336 PMC9978084

[30] Sakurai T Saruta M . Positioning and usefulness of biomarkers in inflammatory bowel disease. Digestion. (2023) 104:30–41. doi: 10.1159/000527846 36404714 PMC9843547

[31] Düzenli T Köseoğlu H Akyol T . NLR and PLR as novel prognostic biomarkers of mucosal healing in ulcerative colitis patients treated with anti-TNF. Inflammation Bowel Dis. (2020) 26:e103. doi: 10.1093/ibd/izaa153 32514550

[32] Benvenuti E Pierini A Gori E Lucarelli C Lubas G Marchetti V . Neutrophil-to-lymphocyte ratio (NLR) in canine inflammatory bowel disease (IBD). Vet Sci. (2020) 7:141. doi: 10.3390/vetsci7030141 32971945 PMC7560079

[33] Akpinar MY Ozin YO Kaplan M Ates I Kalkan IH Kilic ZMY . Platelet-to-lymphocyte ratio and neutrophil-to-lymphocyte ratio predict mucosal disease severity in ulcerative colitis. J Med Biochem. (2018) 37:155–62. doi: 10.1515/jomb-2017-0050 30581352 PMC6294094

[34] Rokkas T Portincasa P Koutroubakis IE . Fecal calprotectin in assessing inflammatory bowel disease endoscopic activity: a diagnostic accuracy meta-analysis. J Gastrointestinal Liver Dis JGLD. (2018) 27:299–306. doi: 10.15403/jgld.2014.1121.273.pti 30240474

[35] Kawashima K Oshima N Kishimoto K Kataoka M Fukunaga M Kotani S . Low fecal calprotectin predicts histological healing in patients with ulcerative colitis with endoscopic remission and leads to prolonged clinical remission. Inflammation Bowel Dis. (2023) 29:359–66. doi: 10.1093/ibd/izac095 35583193

